# A Low-Power All-Digital on-Chip CMOS Oscillator for a Wireless Sensor Node

**DOI:** 10.3390/s16101710

**Published:** 2016-10-14

**Authors:** Duo Sheng, Min-Rong Hong

**Affiliations:** Department of Electrical Engineering, Fu Jen Catholic University, Taipei 24205, Taiwan; bob_hong@princeton.com.tw

**Keywords:** on-chip reference clock generator, wireless sensor node, wireless body area network (WBAN), all digital, low power

## Abstract

This paper presents an all-digital low-power oscillator for reference clocks in wireless body area network (WBAN) applications. The proposed on-chip complementary metal-oxide-semiconductor (CMOS) oscillator provides low-frequency clock signals with low power consumption, high delay resolution, and low circuit complexity. The cascade-stage structure of the proposed design simultaneously achieves high resolution and a wide frequency range. The proposed hysteresis delay cell further reduces the power consumption and hardware costs by 92.4% and 70.4%, respectively, relative to conventional designs. The proposed design is implemented in a standard performance 0.18 μm CMOS process. The measured operational frequency ranged from 7 to 155 MHz, and the power consumption was improved to 79.6 μW (@7 MHz) with a 4.6 ps resolution. The proposed design can be implemented in an all-digital manner, which is highly desirable for system-level integration.

## 1. Introduction

Wireless communication and integrated circuit (IC) technologies continue to develop rapidly; this includes applications in medical services, which extend from closed in-hospital systems to any open-roaming system. Wireless communications immediately convey the physiological data extracted by devices (either worn on or implanted in the body) to the hospital for healthcare and health monitoring. Monitored physiological signals include body temperature, electroencephalograms, and electrocardiography data. The data from a user’s personal device are transmitted to a personal server (such as a smart phone) through the wireless body area network (WBAN). In turn, the server delivers the data to a hospital or medical server by wireless networking [1,2]. The WBAN standards have been established in [3]. There is a growing demand for such biotelemetry applications.

**Figure 1:**
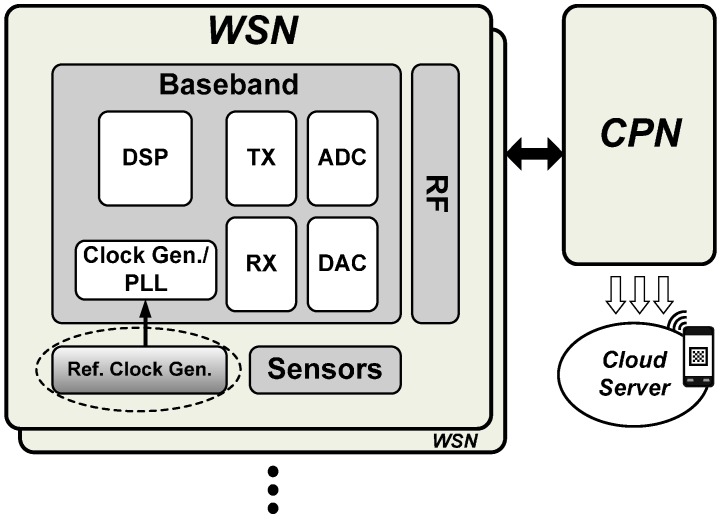
A typical wireless body area network (WBAN) system.

A typical WBAN system is shown in Figure 1. Generally, a WBAN system comprises multiple wireless sensor nodes (WSNs) and a central processing node (CPN). The WSNs sense, filter, and store the physiological signals and wirelessly transmit the data to the CPN and other applications for further processing. According to the requirements for healthcare and health monitoring, there are several important considerations in designing a WBAN system [4]. First, implanted devices are powered by a battery that is not replaceable or rechargeable; hence, to achieve long duration monitoring in biotelemetry applications, the WSN should have ultra-low power requirements [5]. Second, to ensure comfort and noninterference with other bodily functions, medical devices implanted in human bodies should be as small as possible. To reduce the size of the device, its components should be integrated into a single chip. In WBANs, this requires a downsizing of the electronic hardware design from system-on-board (SoB) to system-on-chip (SoC). To further shrink the overall device area, the circuit complexity of the implanted chip should be reduced. Finally, for accurate healthcare and monitoring, the WBAN must provide reliable signal transmission. Thus, especially in the WSN, the quality of the source for the system clock is very important.

To reduce redundant power consumption by the system, WSN designs usually adopt the sleep/wakeup scheme for power management [6]. However, the status change from sleep to wakeup itself consumes power. In addition, a long wakeup time degrades the overall system performance. Thus, reducing the settling time of the clock generator is an important consideration in WSN design. Generally, a clock generator, such as a phase-locked loop (PLL), receives the reference clock signal from an off-chip reference clock generator, such as a crystal oscillator, and generates a system clock signal at the desired frequency for the WSN system. The traditional frequency reference is a tens-of-kilohertz signal generated by a quartz crystal, meaning that the system transits from sleeping to operation in 100 μs or even 1 ms [7,8], which can be as long as the system’s active period. Increasing the reference clock frequency to the megahertz scale would reduce the settling time of the PLL (as the two quantities are inversely proportional) [8] but would also increase the operating power of the reference clock generator, even in the sleeping state. Besides the settling-time problem, quartz crystals are too bulky for easy integration into SoC; consequently, they are unsuitable for WSN in WBAN applications.

Recently, a digitally controlled oscillator (DCO) has been proposed as a reference clock generator for low-power and highly integrated WBAN applications [7]. The output clock frequency of a DCO is controlled by a digital control code. The DCO allows easier porting between different processes and requires lower supply voltage than conventional voltage-controlled oscillators. If a DCO replaces the quartz crystal as the reference clock generator, it can be easily integrated into a WSN, reducing the overall size and hardware costs of the device. Although the DCO is suitable for biotelemetry applications in principle, a further reduction in power consumption, an increase in delay resolution, and an extension of the frequency range are required in practice.

In addition, because a PLL receives the reference clock signal from a reference clock generator, such as the DCO, and generates a system clock signal at the desired frequency for the WSN system. The clock frequency in the WSN system is determined by the DCO output frequency. The ideal reference clock generator can generate any required clock frequency; however, it is hard to be achieved in DCO due to its discrete characteristic. Thus, the DCO resolution should be as high as possible to obtain the suitable clock frequency for WSN system.

This paper proposes a high-resolution, wide-range, low-power reference clock generator for WBAN applications. The cascade-stage structure of the proposed DCO simultaneously achieves high resolution and a wide frequency range. By virtue of the proposed hysteresis delay cells (HDCs), the proposed DCO also achieves a low-frequency output with low circuit complexity and power consumption. In addition, the proposed DCO architecture can be implemented in an all-digital CMOS design manner for cost and power reduction. Thus, it is very suitable for biomedical chip applications and system integration.

The rest of this paper is organized as follows. Section 2 reviews the conventional DCO. The architecture and circuit of the proposed DCO are presented in Section 3. For power and area comparisons, we reconstructed the published approaches and compared them against the proposed HDC in Section 4. Section 5 shows the experimental results and discussions of the proposed design. Finally, the conclusion is given in Section 6.

## 2. Review of Conventional DCOs

Recently, various architectural solutions have been proposed for DCO implementations. The current-starved type DCO [9] alters the delay value by controlling the supply current to the delay cell. Although this DCO has high resolution, it needs a static current source, which increases the static power dissipation. The LC tank DCO [10] also achieves high delay resolution but requires advanced processes and an intensive circuit layout. Both approaches demand complex circuitry, lengthening the design cycle.

To reduce the design cycle when a process or specification is changed, researchers have proposed numerous DCOs from standard cells [11,12,13,14]. Among these, driving capability modulation (DCM) changes the driving current of each delay cell by controlling the number of enabled tristate buffers/inverters [11]. Although the design concept of this approach is straightforward, the linearity and power consumption performances are poor, and the resolution is insufficient. Or-and-inverter (OAI) cells enhance the resolution by different input pattern combinations but do not resolve the poor linearity [12]. Although the digitally controlled varactor (DCV) has a good performance in both resolution and linearity [13], it is difficult to use a few cells to increase the range of operations. Therefore, large power consumption is required because of the many DCV cells needed to maintain an acceptable operational range. To resolve these problems, the hysteresis delay cell (HDC) has been proposed [14]. This HDC consists of several standard cells with delay range as multiple inverters, but the resulting power saving is still limited.

Basically, the conventional logic gate-based DCO is suitable for high-frequency system applications. The period can be straightforwardly extended to middle-to-low frequencies or a wide frequency range by adding more delay units, but this strategy increases the area and leakage current of the device. Alternatively, dividing the frequency output from the high-frequency source is area efficient but restricts the adjustable resolution. This problem can be solved by using a power- and area-efficient delay cell, such as a cascaded hysteresis delay cell (CHDC) [6] or an interlaced hysteresis delay cell (IHDC) [15]. By an advanced process, these hysteresis delay cells achieve low power consumption and a small area. However, the power and area efficiency can be further improved, especially when implemented with normal processes.

As mentioned above, existing DCO architectures are unsuitable for WBAN applications. Thus, the research target of this paper is a DCO that operates as an all-digital reference clock generator in WSNs. To satisfy the system requirements of WBAN, the proposed DCO should feature a low-frequency clock output, low power consumption, wide frequency range, high delay resolution, and low circuit complexity.

## 3. Proposed DCO Design

### 3.1. Architecture

**Figure 2:**
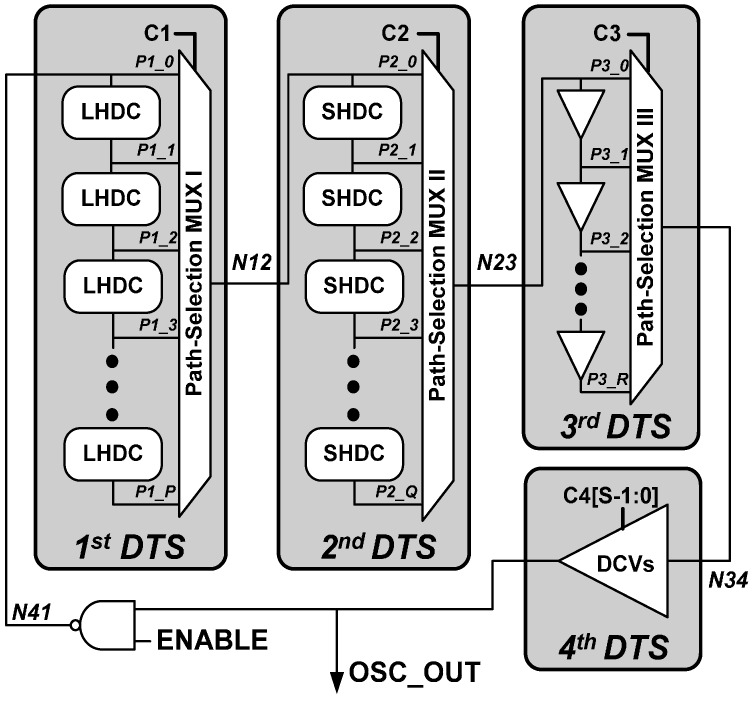
The architecture of the proposed digitally controlled oscillator (DCO).

Figure 2 illustrates the architecture of the proposed DCO, which consists of four delay-tuning stages (DTS’s). It is hard to simultaneously achieve a wide operation range and high delay resolution with the DCO with a single DTS structure. For example, if the delay resolution of DTS is 10 ps, it needs 1000 delay cells to obtain a 10 ns operation range, leading to an increase in area significantly. Thus, the proposed DCO uses a cascading structure that improves the overall delay resolution and extends the operation range.

Each DTS utilizes a different delay cell, the delay resolutions of the DTS’s improve from the 1st DTS (coarsest) to the 4th DTS (finest), and the delay range enlarges from the 4th DTS (narrowest) to the 1st DTS (widest). The first, second, and third DTS’s are configured as path-selection structures to enlarge the tunable delay range. The first delay-tuning stage (the 1st DTS) and second delay-tuning stage (the 2nd DTS) are composed of *P* long hysteresis delay cells (LHDCs) and *Q* short hysteresis delay cells (SHDCs), respectively, where *P* and *Q* denote the number of different delay times provided by the DTS’s. The delay times are obtained by selecting different delay paths organized by the path-selections MUX I in the 1st DTS and MUX II in the 2nd DTS. The details of the delay cell architecture are explained in the next subsection. The 3rd and 4th DTS’s improve the overall delay resolution of the DCO, which is otherwise insufficient for WBAN applications. The 3rd DTS is composed of *R* delay buffers that provide *R* different delay times. The 4th DTS employs *S* DCVs, yielding *S* different delay times at the finest delay resolution.

In each stage of this cascading structure, the controllable range is larger than the delay step of the previous stage. Consequently, the dead zone in this cascading DCO structure never exceeds the least significant bit (LSB) resolution of the DCO.

### 3.2. Circuit Implementation

**Figure 3:**
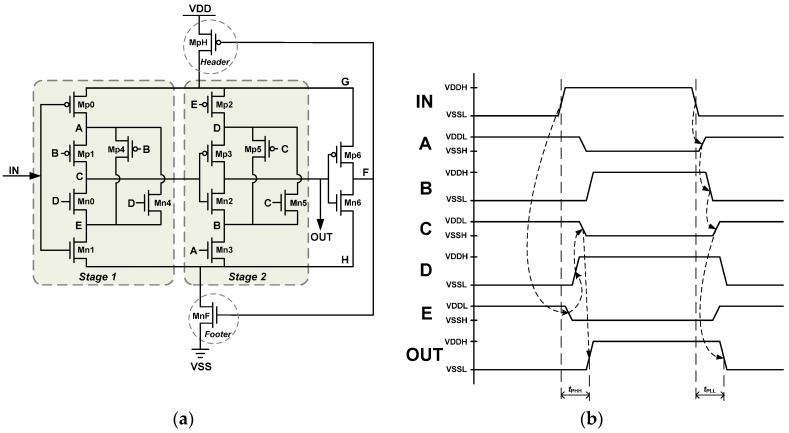
(**a**) The general circuit configurations of the proposed hysteresis delay cell (HDC); (**b**) The operating timing diagrams of the proposed HDC.

The proposed LHDC and SHDC both have hysteresis phenomena, which can induce large delay with low circuit complexity and high power efficiency. Basically, this hysteresis phenomenon is induced by a Schmitt trigger with signal transitions in the interlaced transistors. The general circuit configurations and operating timing diagrams of the proposed HDCs are illustrated in panels (a) and (b), respectively, in Figure 3. The proposed HDC consists of two stages of cascaded transistors and one inverter with a header and a footer transistor. Because of the hysteresis phenomena, the voltage of the internal nodes *G* and *H* when MnF is turned on in the linear region is given by [6,16,17]:

(1) $$V_{G}=\frac{\left[V_{in}-\left|V_{tp}\right|\times (\sqrt{\beta_{MpH}/\beta_{UP}}-1)+V_{DD}\times \sqrt{\beta_{MpH}/\beta_{UP}}\right]}{\sqrt{\beta_{MpH}/\beta_{UP}}+1},$$

(2) $$V_{H}=V_{SS}.$$

Similarly, the voltage of the internal nodes *G* and *H* when MpH is turned on in the linear region can be expressed by

(3) $$V_{G}=V_{DD},\text{ and}$$

(4) $$V_{H}=\frac{\left[V_{in}+\left|V_{tn}\right|\times (\sqrt{\beta_{MnF}/\beta_{DN}}-1)+V_{SS}\times \sqrt{\beta_{MnF}/\beta_{DN}}\right]}{\sqrt{\beta_{MnF}/\beta_{DN}}+1},$$

where *V_tn_* and *V_tp_* are the threshold voltages of nMOS and pMOS, respectively. *β_MpH_* and *β_MnF_* denote the transconductances of the header and footer transistor, respectively. *β_UP_* and *β_DN_* are the equivalent transconductances of the pull-up and pull-down paths in the cascaded transistors, respectively. The header and footer transistors (MpH and MnF, respectively) act as voltage gating cells that scale down the actual supply voltage of the cascaded transistors and confine the short current generated from the internal nodes during voltage transitions, hence increasing the propagation delay.

The cascaded transistors also induce longer delay times than the conventional inverter chain. The delay time is chiefly contributed by Mn0–Mn3 and Mp0–Mp3. Mn4–Mn5 and Mp4–Mp5 connect the temporal floating nodes to a stable state. The delay path is interlaced between these two series of cascaded transistors. In Figure 3b, the original and scaled VDDs (VSS’s) are denoted as VDDH (VSSL) and VDDL (VSSH), respectively.

The proposed HDC operates as follows: When *IN* goes from its initially low state to high, Mn1 is turned on, and *E* goes to VSSH. Subsequently, Mp2 is turned on, and *D* charges to VDDH. Mn0 is then turned on, and *C* is discharged to VSSH. *C* turns on Mp3 and charges *OUT* to VDDH. In summary, as *IN* goes from low to high, the rising transition of the *IN* signal propagates through Mn1, Mp2, Mn0, and Mp3 to *OUT*. Similarly, the falling transition of the *IN* signal propagates through Mp0, Mn3, Mp1, and Mn2 to *OUT*. By this mechanism, the proposed HDC achieves higher area- and power-efficiency than conventional delay cells.

The propagation delay of the proposed HDC can be divided into *t*_PHH_ (*IN* = rising edge to *OUT* = rising edge) and *t*_PLL_ (*IN* = falling edge to *OUT* = falling edge), as shown in Figure 3b. The propagation delay time *t*_PHH_ is contributed by Mn1, Mp2, Mn0, and Mp3 as expressed by

(5) $$t_{PHH}=t_{PHL\_Mn1}+t_{PLH\_Mp2}+t_{PHL\_Mn0}+t_{PLH\_Mp3}.$$

Each MOS propagation delay time can be calculated by the average current (*I_av_*), the load capacitance (*C_load_*), and the voltage swing [18]. For example, the propagation delay of Mn1 can be expressed by

(6) $$t_{PHL\_Mn1}=\frac{C_{load}\times \left(Vdd-V_{H}\right)}{2\times I_{av}},\text{ and}$$

(7) $$I_{av}=\frac{1}{2}\left\{\left[\frac{1}{2}k_{n}{\left(Vdd-V_{H}-V_{tn}\right)}^{2}\right]+k_{n}\left[\left(Vdd-V_{H}-V_{tn}\right)\left(\frac{Vdd-V_{H}}{2}\right)-\frac{1}{2}{\left(\frac{Vdd-V_{H}}{2}\right)}^{2}\right]\right\}.$$

The other three propagation delay times can be calculated in a similar manner. In addition, the propagation delay time *t*_PLL_ can be also calculated in a similar manner. As a result, according to the above equations, the propagation delay of the proposed HDC can be formulated by *V_G_* and *V_H_*.

**Figure 4:**
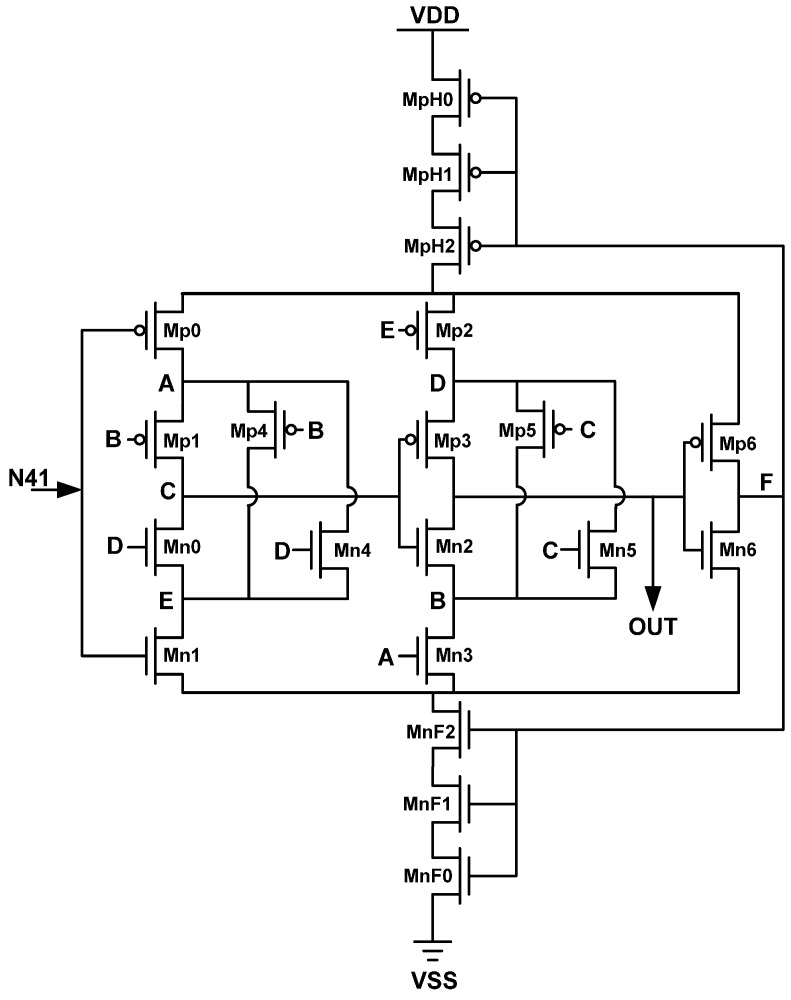
The circuit diagrams of the short hysteresis delay cell (SHDC).

**Figure 5:**
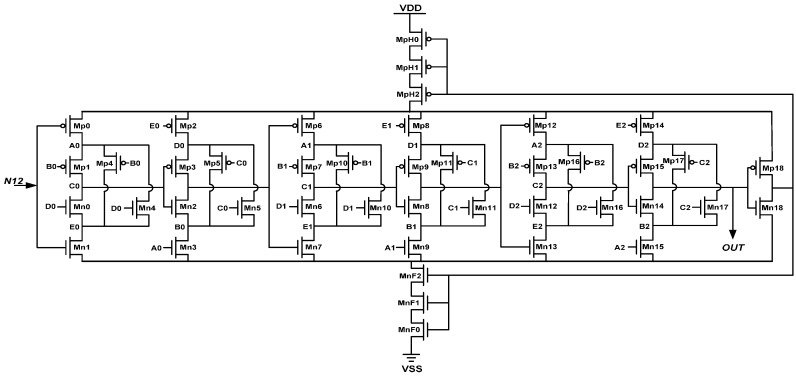
The circuit diagrams of the long hysteresis delay cell (LHDC).

The design concept of the proposed HDC enables further delay of the HDC; the delay of HDC can be further extended as the number of the cascaded transistor series and the header/footer transistor increases. Figure 4 and Figure 5 show the circuit diagrams of the LHDC in the 1st DTS and the SHDC in the 2nd DTS, respectively.

The 3rd DTS is configured as a path-selection structure that is the same as the 1st and 2nd DTS’s, and the only difference is that the 3rd DTS is composed of simple buffers (two-inverter chain) where the delay time is smaller than the LHDC and the SHDC in the previous DTS’s.

**Figure 6:**
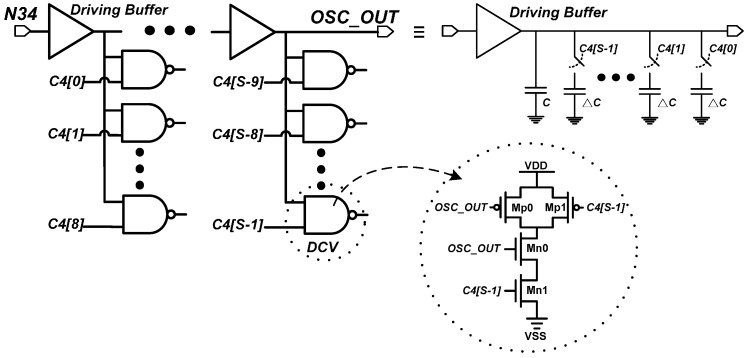
The circuit diagrams of the 4th delay-tuning stage (DTS).

To improve the delay resolution, the proposed 4th DTS includes a DCV with a changeable gate capacitance. Under digital control code *C4* and different output loadings of the driving buffer, the slight changes in the gate capacitance alter the delay of the 4th DTS. The DCV is a two-input NAND gate as illustrated in Figure 6 [14]. The total gate capacitance of transistors Mn0 and Mp0 varies with *C4* input states. The programmable delay (Δ*T*) of a DCV in different *C4* states can be calculated easily using the following linear equation:

(8) ΔT=K×ΔC,

where *K* denotes the delay factor of driving buffer, and ΔC is the capacitance difference between different *C4* states (*C4* = 1 and *C4* = 0). For example, according to Equation (8), as the *K* value of the driving buffer is 2.3 (ns/pF), and Δ*C* is around 2 fF, then Δ*T* of 4.6 ps is obtained. Consequently, the overall delay resolution of the DCO is improved from several hundred picoseconds to several picoseconds.

## 4. Delay Cell Comparisons

For power and area comparisons, we reconstructed the published approaches that provide large delay times under TSMC 0.18 μm CMOS standard technology and compared them against our proposed HDC in HSPICE simulations. The reconstructed delay cells were the CHDC type (Approach I) [6] and the IHDC type (Approach II) [15]. For a fair comparison, the delay cells were configured as typical ring oscillators with similar output frequencies. Because the silicon area of the published delay cells including CHDC [6] and IHDC [15] is not available in the published paper, we use the transistor count to evaluate the hardware cost (area). The definition of area efficiency is how many transistors is taken to generate the specified output frequency. Table 1 summarizes the delay comparisons simulated at 1.8 V and a typical corner case.

**Figure 7:**
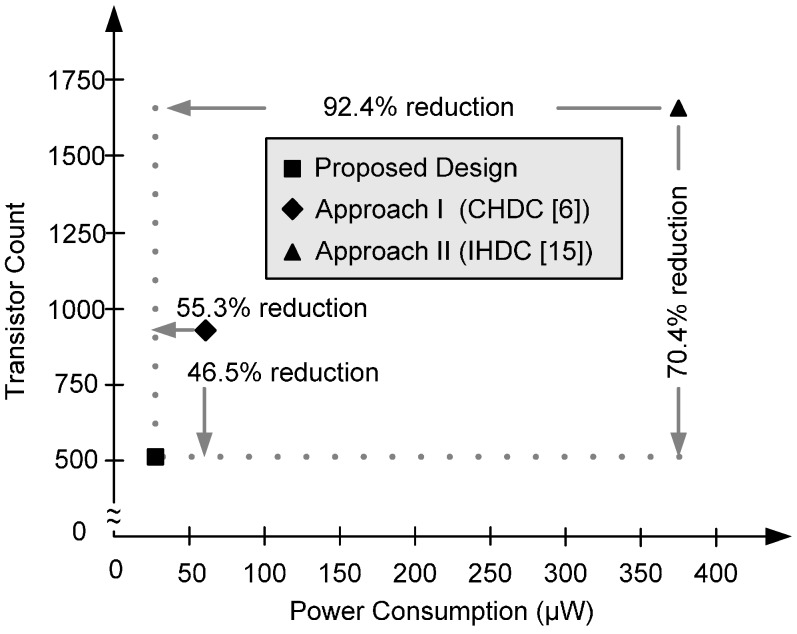
The power consumption and area comparisons of different delay cells.

In terms of hardware cost, the proposed HDC, Approach I, and Approach II required 528, 928, and 1660 transistors, respectively. Therefore, compared with Approaches I and II, our proposed approach reduces the transistor count (which largely contributes to hardware costs) by 46.5% and 70.4%, respectively. Moreover, the proposed HDC also has the lowest power consumption as compared with the other designs; the power reduction ratios are 55.3% and 92.4%, as compared with Approach I and Approach II, respectively. Figure 7 also shows that our proposal has significant improvement in power consumption and area as compared with the other designs. In summary, the proposed structure provides a better power-to-delay ratio than other HDC type structures, implying that the proposed delay cell more effectively conserves power and area for a given delay.

## 5. Experimental Results and Discussions

**Figure 8:**
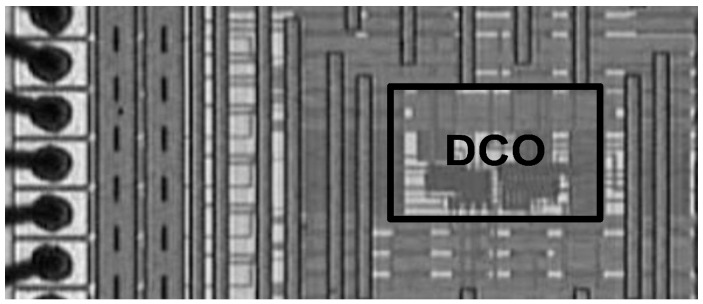
The chip microphotograph of the DCO chip.

On the basis of the requested frequency range and resolution of WBAN applications, the design parameters of the proposed DCO were determined as *P* = 10, *Q* = 4, *R* = 25, and *S* = 56. To verify the feasibility and performance of the proposed DCO, a test chip was fabricated in a TSMC 0.18 μm 1P6M CMOS process with an area of 10,787 μm^2^, where the chip microphotograph of the DCO chip is shown in Figure 8.

**Figure 9:**
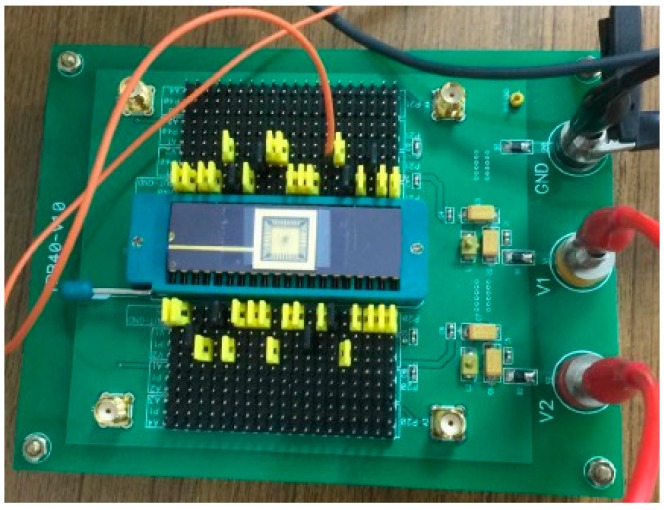
The testing platform of the DCO chip.

**Figure 10:**
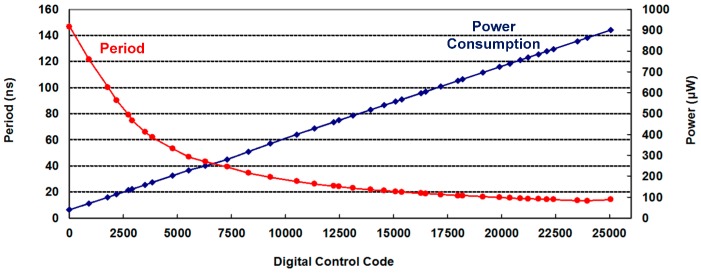
The measured output periods and power consumptions under different digital codes.

**Figure 11:**
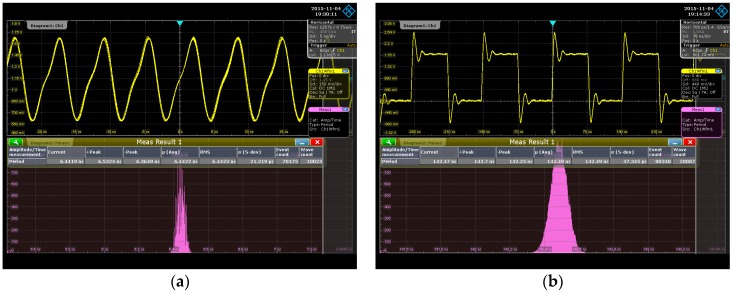
Jitter histogram at (**a**) 155 MHz and (**b**) 7 MHz.

To test the performance of the DCO, the DCO output signal was measured using an R&S RTO1044 oscilloscope at 1.8 V/25 °C. The testing platform is shown in Figure 9. Table 2 lists the least delay step and operation range of each tuning stage in the proposed DCO. As the controllable range of each tuning stage is larger than the smallest DCO delay step of the previous stage, the functionality of the device is guaranteed. The average DCO resolution was 4.6 ps. The measured output periods and power consumptions under different digital codes are plotted in Figure 10. The proposed DCO clearly demonstrates period-to-code linearity. Figure 11a,b shows that the root-mean-square (RMS) jitter is 21.22 ps at 155 MHz with 916.2 μW and 47.35 ps at 7 MHz with 79.56 μW, respectively.

Table 3 compares the results of the proposed design and state-of-the-art DCOs. Because only the proposed DCO and the DCO in [6] provide output frequencies below 10 MHz, these two designs alone meet the requirements of on-chip oscillator applications. In addition, our proposed design exhibits superior LSB resolution and RMS jitter performances than the designs in [18] and [19]. Furthermore, because these designs are implemented by different processes and operated at different supply voltages, their power consumptions and areas are difficult to compare directly. Thus, for a fair comparison, the delay cells of [6] and [15] were rebuilt under the same technology as mentioned in Section 4. Although these published designs are implemented by a more advanced process than the proposed DCO in this paper, the proposed DCO can achieve a good delay resolution, jitter, and operation range compared with these designs. In summary, the proposed low-power solution improves the resolution and lowers the output frequency and jitter without compromising the performance.

## 6. Conclusions

In this paper, we have proposed an all-digital and low-power on-chip reference clock generator for WBAN applications. The proposed hysteresis delay cell further reduces the power consumption and hardware costs by 92.4% and 70.4%, respectively, relative to conventional designs. In measurements, the operational frequency range was 7–155 MHz, and the power consumption was improved to 79.6 μW (@7 MHz) with a 4.6 ps resolution. The proposed design not only reduces the power consumption but also improves the LSB resolution and delay linearity of the DCO. Moreover, the proposed reference clock generator can be implemented in an all-digital manner, which is beneficial for system-level integration. In summary, this paper offers an area- and power-efficiency clock source in low-power WBAN applications.

## Figures and Tables

**Table 1:** Power and area comparisons with different delay cells.

Approach	Frequency (MHz)	Power (μW)	Transistor Count
Proposed HDC	9.8	28.4	44 × 12 = 528
CHDC [6]	9.8	63.5	40 × 23 = 920
IHDC [15]	10	375	20 × 83 = 1660

**Table 2:** Measurement results of step/range of tuning stage.

	Range (ns)	Step (ns)
1st DTS	111.69	11.169
2nd DTS	18.66	4.665
3rd DTS	6.436	0.257
4th DTS	0.26	0.0046

**Table 3:** Performance comparisons.

Performance Indices	Proposed DCO	CHDC [6]	IHDC [15]	TCASII‘11 [19]	TCASII‘08 [20]
Process	0.18 μm CMOS	90 nm CMOS	90 nm CMOS	65 nm CMOS	0.13 μm CMOS
Supply Voltage (V)	1.8	1	1	1	1.28
Operation Range (MHz)	7~155	3.4~163.2	180~530	47.8~538.7	300~1300
LSB Resolution (ps)	4.6	2.05	3.5	17.4	12
Power Consumption (μW)	916.2 @155 MHz	166 @163.2 MHz	190.1 @480 MHz	205 @481.6 MHz	4480 @950 MHz
	79.6 @7 MHz	5.4 @3.4 MHz	105 @200 MHz	142 @58.7 MHz	
RMS Jitter (% of Period)	21.22 ps @155 MHz (0.33%)	49.3 ps @5 MHz (0.02%)	N/A	13.2 ps @64.49 MHz (8.51%)	10.4 ps @950 MHz (1.09%)
	47.35 ps @7 MHz (0.03%)				
Area (μm^2^)	10,787	6400	N/A	10,000	7500
